# Understanding Hyperuricemia: Pathogenesis, Potential Therapeutic Role of Bioactive Peptides, and Assessing Bioactive Peptide Advantages and Challenges

**DOI:** 10.3390/foods12244465

**Published:** 2023-12-13

**Authors:** Yanchao Chen, Jing Yang, Qinchun Rao, Chen Wang, Xiaoyong Chen, Yu Zhang, Huayi Suo, Jiajia Song

**Affiliations:** 1 College of Food Science, Southwest University, Chongqing 400715, China; 2 Chongqing Engineering Research Center for Processing & Storage of Distinct Agricultural Products, Chongqing Technology and Business University, Chongqing 400067, China; 3 Department of Health, Nutrition, and Food Sciences, Florida State University, Tallahassee, FL 32306, USA

**Keywords:** hyperuricemia, proteins, bioactive peptides, mechanisms

## Abstract

Hyperuricemia is a medical condition characterized by an elevated level of serum uric acid, closely associated with other metabolic disorders, and its global incidence rate is increasing. Increased synthesis or decreased excretion of uric acid can lead to hyperuricemia. Protein peptides from various food sources have demonstrated potential in treating hyperuricemia, including marine organisms, ovalbumin, milk, nuts, rice, legumes, mushrooms, and protein-rich processing by-products. Through in vitro experiments and the establishment of cell or animal models, it has been proven that these peptides exhibit anti-hyperuricemia biological activities by inhibiting xanthine oxidase activity, downregulating key enzymes in purine metabolism, regulating the expression level of uric acid transporters, and restoring the composition of the intestinal flora. Protein peptides derived from food offer advantages such as a wide range of sources, significant therapeutic benefits, and minimal adverse effects. However, they also face challenges in terms of commercialization. The findings of this review contribute to a better understanding of hyperuricemia and peptides with hyperuricemia-alleviating activity. Furthermore, they provide a theoretical reference for developing new functional foods suitable for individuals with hyperuricemia.

## 1. Introduction

As the by-product of human purine metabolism, uric acid (UA) is mainly synthesized endogenously by the liver, intestines, and other tissues. It is also obtained from an exogenous source primarily constituted of animal proteins. Elevated production or reduced excretion of UA can lead to higher-than-normal serum uric acid (SUA) levels, inducing hyperuricemia (HUA). Currently, HUA is becoming more prevalent. According to a survey conducted in China, the prevalence of HUA from 2018 to 2019 was 14%, with men and women experiencing rates of 24.4% and 3.6%, respectively. Similarly, 20% of adults in the United States have HUA [1,2]. There is strong evidence that HUA may increase the chance of developing gout and chronic kidney disease, obesity, diabetes, and hypertension [3].

Drugs are still the primary method of treatment for HUA nowadays. Allopurinol is the most commonly used drug to decrease SUA levels. The structural analogs of hypoxanthine and xanthine are allopurinol and its metabolite oxypurinol. They can combine with it to inhibit the activity of xanthine oxidase (XO) to reduce the synthesis of UA. Probenecid, another drug, primarily decreases UA reabsorption by urate transporter 1 (URAT1) activity inhibition, increasing UA kidney clearance. Allopurinol frequently causes gastrointestinal discomfort and varying degrees of rash, and febuxoat, a new XO inhibitor, is easily able to induce cardiovascular disease [4,5,6]. These are just a few examples of drugs with specific side effects. Dietary management is another method of treating HUA. This method lowers SUA levels by decreasing the consumption of exogenous purines, i.e., foods high in purines, particularly hypoxanthines. Since tasty foods typically contain more purines (such as umami components), long-term dietary restriction to relieve HUA is impractical [7]. Moreover, studies have shown that bioactive ingredients can alleviate HUA, such as flavonoids, phenolic acids, alkaloids, polysaccharides, and saponins. These active substances alleviate HUA by inhibiting UA synthesis and promoting UA excretion, and some can also improve the inflammatory response caused by HUA [8]. For instance, quercetin alters the intestinal flora of HUA chickens, significantly increasing the abundance of *Lactobacillus aviarius*. This increase promotes purine nucleoside degradation, thereby reducing SUA levels [9]. Ferulic acid can downregulate the mRNA expression level of XO and bind to the active site of XO through hydrogen bonding and hydrophobic interaction, thereby reducing UA production to alleviate HUA [10]. *Eurycoma longifolia* alkaloid reduces UA production by decreasing the protein expression levels of phosphoribosyl pyrophosphate synthetase (PRPS) and hypoxanthine phosphoribosyltransferase (HPRT) in the liver. Additionally, it restores the expression levels of organic anion transporter 1 (OAT1) and ATP-binding transporter G family member 2 (ABCG2) in the kidney to promote UA excretion [11]. Guluronic oligosaccharides alleviate HUA through multiple mechanisms, including restoring normal expression levels of XO and adenosine deaminase (ADA), regulating UA transporter expression at the mRNA level, and altering gut microbiota composition [12]. Quinoa bran saponins inhibit XO and ADA activities and also regulate UA transporter expression at the mRNA level to alleviate HUA [13]. In addition, protein hydrolysates from food sources have also received extensive attention in recent years, exhibiting a variety of biological effects and providing a new way to treat HUA.

Enzymatic proteolysis (gastrointestinal digestion or protease hydrolysis), hydrolysis by chemical reaction using acid or alkaline reagents, or fermentation, are all methods for releasing food protein-derived peptides. These peptides can be obtained from animals, plants, fungi, microorganisms, and their products [14]. A wide range of food protein-derived peptides have demonstrated various biological activities. For instance, six new antioxidant peptides have been identified in pearl shell meat hydrolysate, showcasing potential for use as natural materials in the development of functional foods [15]. The anti-inflammatory peptide derived from walnut meal protein is anticipated to function as a natural anti-inflammatory active substance in the pharmaceutical industry [16]. A hexapeptide, recognized as the most effective inhibitor of angiotensin I-converting enzyme in silver carp fish seed protein hydrolysate, may find applications in functional foods or as a natural antihypertensive drug [17]. Several novel bioactive peptides with anti-hypercholesterolemic activity have been isolated from quinoa protein, offering potential as adjuvant therapy for hypercholesterolemia [18]. Additionally, a pentapeptide with α-glucosidase inhibitory activity has been discovered in rice bran fermentation broth, providing a new avenue for the development of anti-diabetic drugs [19]. Studies have been conducted over the past few years on the isolation of peptides with anti-hyperuricemia bioactivity from animal and plant food sources, with the majority of these studies concentrating on marine fish [20,21,22,23]. To the best of our knowledge, there is no comprehensive, systematic evaluation of protein peptides from foods that alleviate HUA.

The purpose of this review is to elucidate the pathogenesis of HUA and to summarize the types, mechanisms of action, advantages, and challenges of food protein-derived peptides that can alleviate HUA. The findings of this review help us comprehend the therapeutic potential of anti-hyperuricemia peptides and give us a theoretical foundation for their application in various industries, including food and medicine.

## 2. Pathogenesis of HUA

### 2.1. Excessive UA Production

The main source of UA in vivo is the degradation of endogenous nucleic acids, and defects in nucleic acid metabolic pathways may increase the risk of HUA [24]. PRPS hyperactivity and HPRT deficiency both increase endogenous purine production, leading to overproduction and accumulation of UA [3]. PRPS1 is responsible for catalyzing ribose 5-phosphate to phosphoribosyl pyrophosphate. Excessive PRPS1 activity increases the amount of purine nucleotide synthesis, resulting in elevated purine content in the body and an increase in UA levels beyond the normal range [25]. HPRT is a transferase that catalyzes the conversion of hypoxanthine to inosine monophosphate (IMP) and guanine to guanosine monophosphate (GMP). Partial HPRT deficiency can result in increased purine content in the body, inducing HUA, which is a potential cause of familial juvenile gout [26].

**Figure 1:**
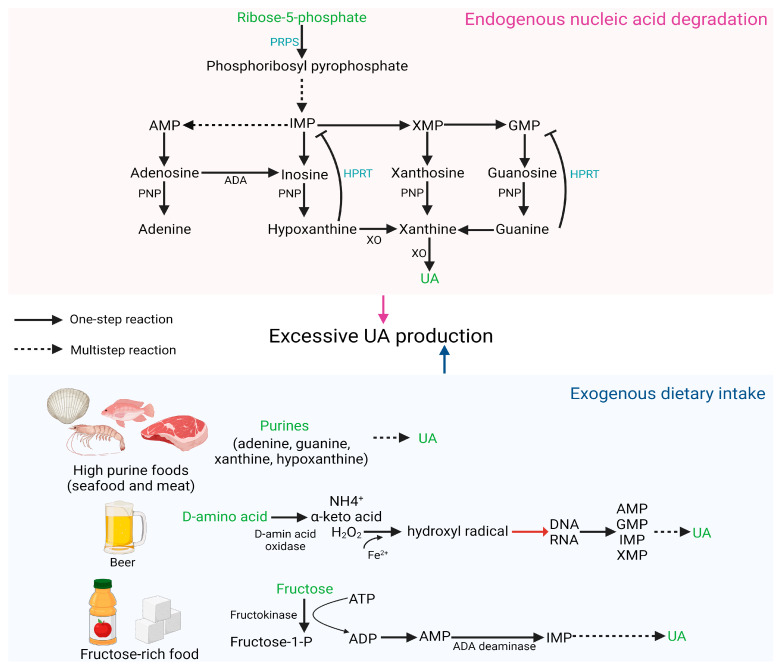
HUA occurs due to excessive synthesis of UA.

Diet plays a crucial role in the pathogenesis of abnormal SUA levels and associated complications. Excessive consumption of certain foods, such as meat, seafood, beer, and those high in fructose, may elevate the risk of HUA [27]. Purines (adenine, guanine, hypoxanthine, and xanthine) are commonly found in foods, particularly in seafood and meat. Numerous studies have demonstrated that prolonged high intake of meat and seafood can result in an increase in exogenous purines, leading to elevated SUA concentration and increased incidence of HUA [28,29,30]. D-amino acid oxidase catalyzes the formation of D-amino acids into H_2_O_2_, which is further oxidized to hydroxyl radicals in the presence of Fe^2+^. Hydroxyl radicals cause DNA damage and generate numerous purine bases, subsequently oxidized to UA by various enzymes. Due to the high content of D-amino acids in beer, excessive beer consumption can elevate SUA levels and increase the likelihood of developing gout [31]. Excessive fructose intake accelerates the degradation of purines, further heightening the risk of HUA. The catalysis of fructose by fructokinase to fructose-1-phosphate results in the degradation of ATP into AMP, lowering the phosphoric acid level in the cell. This increases the activity of AMP deaminase, accelerates the conversion of AMP to IMP, and ultimately results in an overabundance of UA synthesis [32]. Figure 1 depicts HUA produced as a result of excessive UA production.

### 2.2. Reduced UA Excretion

**Figure 2:**
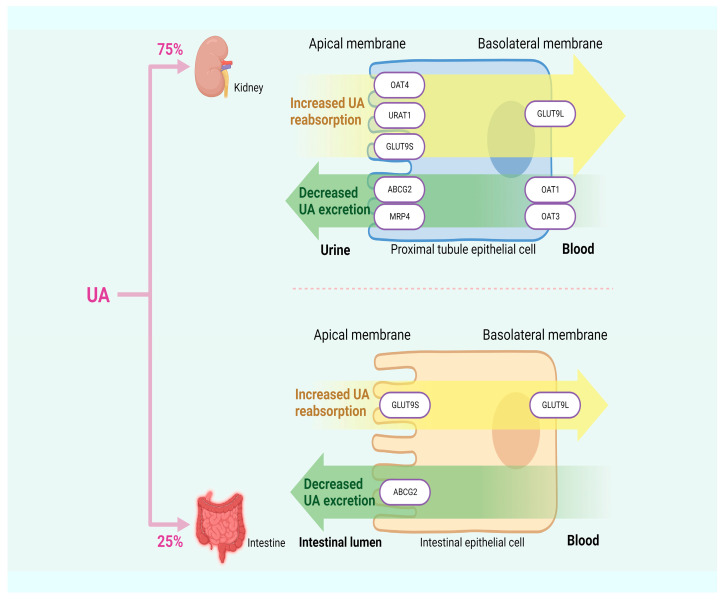
HUA arises from inadequate excretion of UA.

About 10% of HUA is attributed to excessive UA synthesis, while the remaining 90% results from insufficient UA excretion. Approximately 75% of UA in humans is excreted by the kidneys, with the remaining 25% is excreted by the intestines [33,34]. UA transporters play a crucial role in the process of UA excretion and can be classified into two groups based on their functions: UA reabsorption transporters, such as URAT1, OAT4, and glucose transporter 9 (GLUT9); and UA excretion transporters, such as OAT1, urate transporter (UAT), multidrug resistance-associated protein 4 (MRP4), ABCG2, and sodium-dependent phosphate transporter. Dysfunction in these transporters can lead to insufficient UA excretion, thereby inducing HUA [33]. In Figure 2, HUA results from insufficient UA excretion.

URAT1, located in the apical membrane of the renal proximal tubules and serving as a significant target for anti-hyperuricemic drugs, is responsible for transporting UA from the lumen of proximal tubules to the renal tubular epithelial cells. Previous studies have demonstrated a positive correlation between SUA content and URAT1 expression, providing further support for the idea that URAT1 enhances the reabsorption of UA by the renal tubules, thereby increasing SUA content [35,36]. OAT4, along with OAT1, OAT2, OAT3, and URAT1, belongs to the multispecific transporter family. The reabsorption of urinary organic anions, such as UA, back into the proximal renal tubule cells is mediated by OAT4, which is expressed in the apical membrane of these cells [37]. GLUT9 not only reabsorbs UA from the lumen of the renal tubules and participates in renal UA reabsorption but also expresses in the liver and intestine. The first 29 residues of the N-terminal domain of the two isoforms of GLUT9, GLUT9L, and GLUT9S, are the only differences between them. GLUT9L is primarily located in the basal membrane of the proximal renal tubules, while GLUT9S is found in the apical membrane. Although downregulating the expression level of the GLUT9 gene in the liver can promote UA excretion through urine, GLUT9 in the kidney may still maintain intact UA reabsorption activity, ultimately resulting in SUA content higher than the normal range [38,39,40].

OAT1 and OAT3 play a role in secreting organic anions (including UA) in the kidneys into proximal tubular cells through the basolateral membrane. UA accumulates in the body due to reduced expression levels of OAT1 and OAT3, which are indicated by lower UA content in urine. This suggests that both are involved in renal UA secretion [41,42]. MRP4 is primarily located at the apical membrane of proximal renal tubule cells, facilitating the excretion of UA and other intracellular organic anions into the urine [43]. A UA efflux transport protein called ABCG2 can be found in the kidney and intestine. When it malfunctions, it can either create excessive UA in the kidney due to the obstruction of UA excretion in the intestine or block the process of renal UA excretion, resulting in increased SUA levels. According to another finding, ABCG2 dysfunction decreases intestinal UA excretion, which increases SUA levels [44,45].

## 3. Food-Derived Protein Peptides in the Treatment of HUA

### 3.1. XO Inhibitory Peptides

The 290 kDa homodimer XO has an independent catalytic activity for each monomer. Each monomer contains a molybdenum molybdopterin (Mo-pt), a flavin adenine dinucleotide, and two different [2Fe−2S] centers [46]. The active cavity reaches the Mo-pt center, the substrate oxidation site, a long, narrow channel. Circular dichroism spectra of the luteolin–XO system have been used in a previous study to demonstrate how the secondary structure of the protein changes as the molar ratio of luteolin to XO increases. It showed a decrease in the content of β-sheet, random coil, and β-turn and an increase in the α-helix structure. As a result, the active site of XO is more challenging to develop and less likely to bind to the substrate xanthine, which ultimately decreases the catalytic activity of XO [47].

Additionally, bioactive peptides can bind to XO active sites. By doing so, they can change the spatial structure of the active sites and prevent substrates from entering them. This inhibits XO’s activity and lowers UA production [47,48,49]. Table 1 lists the XO inhibitory peptides from different food sources.

#### 3.1.1. Marine Organisms

Marine ecosystems contain more than half of the world’s biota, and the wide variety of marine life offers a rich source for the development of bioactive compounds, including peptides [50]. ICRK, FDAK, and MMER, three different types of peptides with potent XO activity inhibition, have been isolated from *Thunnus orientalis*. The mechanism of action of MMER and FDAK is similar to that of ICRK, which blocks xanthine from binding to XO active sites by occupying the entrance of the hydrophobic channel [51]. Using an ultrafiltration membrane for sequential separation and concentration of tuna hydrolysate, the peptide ACECD—which can be inserted into the Mo-pt center of XO—has been discovered from 600–1000 Da components. In addition, it is attached to the amino acid residues close to the XO active cavity, which prevents xanthine from being oxidized and reduces the catalytic activity of XO. As a result, ACECD may be effective as a safe XO inhibitory drug to alleviate HUA [21]. After virtual screening and molecular docking, the tetrapeptide EEAK has been identified as having the most significant interaction with the critical XO amino acid residues. The inhibition rates of the synthesized EEAK on XO are 32.3%, 51.3%, and 73.1%, respectively, at the doses of 0.25, 0.60, and 1.00 mg/mL, indicating potential XO inhibitory activity, which may be the result of EEAK altering the structure of XO. It is important to note that electrostatic interaction and hydrogen bonding are key factors in the ability of EEAK to inhibit XO activity [52]. The hydrolyzate of *Decapterus maruadsi* contains XO inhibitory peptides KGFP, FPSV, FPFP, and WPDGR, which have been isolated and identified. They show substantial XO inhibitory activity due to the π-π stacking interaction between the Phe in the synthesis of tetrapeptides (FPSV and FPFP) and the Phe914 of XO, which is consistent with the findings of previous research [53,54]. XO inhibitory peptides PDL and SVGGAL, with half maximal inhibitory concentration (IC_50_) values of 4.37 ± 0.11 mg/mL and 5.59 ± 0.09 mg/mL, respectively, have been obtained from *Auxis thazard* by enzymatic hydrolysis and purification. The formation of hydrogen bonds, hydrophobic interactions, and van der Waals forces in the XO amino acid residues is mostly responsible for this biological activity of these two peptides [55]. Small yellow croaker hydrolyzate was used to screen the peptides WDDMEKIW and APPERKYSVW, with hydrogen bonds and hydrophobic interactions being crucial in the formation of stable peptide–XO complexes [56]. The interaction mechanism between peptides and XO was disclosed by molecular docking of the XO inhibitory peptides from oyster hydrolyzate, namely ALSGSW, GGYGIF (GF6), and MAIGLW. Next, the GF6 amino acid sequence with the highest XO inhibitory activity has been improved. The findings demonstrated that substituting the N-terminal Gly of GF6 with Trp may significantly decrease XO activity and that peptides produced by simple structured amino acids and aromatic amino acids (especially Trp) alternately have better XO inhibitory effect in vitro [57]. *Auxis thazard* oligopeptides (ATO) have been obtained by enzymatic hydrolysis, and the fraction (<1 kDa) exhibits the strongest XO inhibitory activity (IC_50_ value, 11.23 ± 0.31 mg/mL) [58].

The enzymatic hydrolysates of Pacific white shrimp have been used to identify XO inhibitory peptides AEAQMWR, EFGMGGW, and AGGINLAR. The most potent XO inhibitory activity was demonstrated by the peptide AEAQMWR, which formed a hydrogen bond with the Mos3004 active site of XO. When 2 mM of peptides and 0.1 mM of allopurinol were used to culture HK-2 cells, the amount of UA in the cells was discovered to be significantly lower. Among these, AEAQMWR and AGGINLAR displayed inhibitory effects on XO comparable to those of allopurinol, making them candidates for treating HUA [59]. Seven XO inhibitory peptides, namely YNITGW, GDEY, AGDY, PDARG, YGDE, VTGW, and EDDDA, have been virtually screened from the entire protein sequence of Pacific white shrimp. These peptides can bind to the active site of XO, thereby inhibiting its activity. In HK-2 cells, YNITGW reduces UA content in a concentration-dependent manner. It is noteworthy that the UA reduction effect of 3 mM of YNITGW is comparable to that of 0.1 mM of allopurinol. Different positions in the YNITGW sequence were explored by inserting Trp, revealing that a peptide containing two Trp exhibits stronger inhibitory activity, while the peptide WYNITGWW with three Trp shows a lower inhibitory effect on XO. This suggests that Trp content is closely related to the activity of XO inhibitory peptides [60].

According to a study, bonito hydrolysates (BH) effectively block the rat XO enzyme to produce high levels of anti-hyperuricemic activity. From BH, the peptides WML and PGACSN were purified and identified. They were subsequently chemically synthesized, and their XO inhibitory activity was confirmed. The hydrophobic interaction that makes it simpler for WML to enter the active site of XO causes it to have a higher inhibitory activity on XO than PGACSN does at the same concentration (20 mM) [20]. Tuna protein hydrolysates (TPH) were administered to HUA mice, and the SUA levels were found to be decreased. An FH dipeptide from the alcohol-soluble portion of TPH was found to have the highest XO inhibition (21.69 ± 1.12%). The inhibition of XO activity occurs due to the formation of hydrogen bonds with Ser876 and Thr1010 in XO and π-π stacking interactions with Phe914. These interactions occupy the hydrophobic channel of XO, preventing substrate binding to active sites. Consequently, di/tripeptides containing Phe may serve as potent XO inhibitors against HUA [54]. Mice with HUA were administered varying doses of ATO, with the high-dose ATO group (600 mg/kg) demonstrating a liver XO inhibition effect similar to that of allopurinol. This suggests that ATO is an effective XO inhibitor [58].

#### 3.1.2. Ovalbumin

Ovalbumin is the most abundant protein in egg whites, accounting for about 54% of the total, and can be regarded as a good source for developing bioactive peptides [61]. Virtual hydrolysis of ovalbumin was used to screen out XO inhibitory peptidesEEK, TNDC, EGK, EER, and DNEC, and their biological activities were later confirmed through in vitro synthesis. The XO inhibition rate of EEK was much higher than that of other peptides at a concentration of 0.50 mg/mL at 73.67%. The results of molecular docking revealed that EEK binds to the active center of XO through a variety of interactions, including carbon–hydrogen bonds, salt bridges, conventional hydrogen bonds, and attractive charge interactions. Among these interactions, Glu802, Phe1009, and Arg880 of XO are key residues in the peptide–XO interaction [62].

#### 3.1.3. Milk

Milk protein-derived peptides possess numerous beneficial health properties, making them suitable for the production of nutritional health products and functional foods. They have garnered widespread attention globally [63]. A study has identified XO inhibitory peptides from whey protein after simulating gastrointestinal digestion. Subsequently, PEW and LLW, both displaying the strongest XO inhibitory activity, were synthesized. These peptides form stable complexes with key amino acids of XO through hydrogen bonds and hydrophobic interactions, preventing the substrate from binding to XO [64]. Four novel XO inhibitory peptides, GL, PM, AL, and AM, have been identified from whey protein, exhibiting strong XO inhibitory activity in vitro. Molecular docking results revealed that these peptides bind to XO through hydrogen bonding, hydrophobic interactions, and van der Waals forces. They can serve as XO inhibitors for the prevention and treatment of HUA [65]. With IC_50_ values of 0.28 and 0.13 mg/mL, respectively, α-lactalbumin and casein hydrolysates made with alkaline protease have the strongest inhibitory effect on XO. Eight peptides with XO inhibitory activity were eliminated using gel filtration chromatography and LC-MS/MS, including VYPFPGPI, GPVRGPFPIIV, VYPFPGPIPN, VYPFPGPIHN, QLKRFSFRSFIWR, LVYPFPGPIHN, AVFPSIVGR, and GFININSLR. These peptides were synthesized in vitro, and tests were done to confirm their biological activity. GPVRGPFPIIV is the one with the strongest activity (IC_50_ value, 4.67 mM), and VYPFPGPIHN is the one that had the lowest activity (IC_50_ value, 8.02 mM). Because these peptides mostly establish hydrogen bonds with XO residues (Ser774, Glu879, His879, Ser710, and Lys771), molecular docking suggests that peptides can maintain a stable binding state with XO [66]. According to research, Trp, which is similar in structure to xanthine (both with C6 and C5 ring structures), is partially responsible for the inhibitory effect of bioactive peptides on XO activity. Both individual Trp and dipeptides containing Trp have the same inhibitory effect on XO activity; however, the location of Trp within the dipeptide has no bearing on XO inhibitory activity. Trp can be found in milk protein. Therefore, XO inhibitors can be made using Trp and Trp-containing dipeptides produced during hydrolysis [67].

SUA levels decrease significantly in a dose-dependent manner following gavage treatment of HUA mice with whey protein hydrolysates (WPH). It is noteworthy that SUA levels in the 400 mg/ kg WPH medium-dose group and the 800 mg/kg WPH high-dose group are nearly identical to those in the standard group; the lowered XO activity in serum and liver is a good explanation for this occurrence [68].

#### 3.1.4. Spider Venom

Spider venom is composed of various bioactive substances, including small ions, peptides, and proteins, with antibacterial, anti-inflammatory, and other functions [69]. A short peptide, NCTX15, with the sequence QSGHTFK, has been identified from the *Nephila clavata* toxin gland. An analysis revealed that NCTX15 exhibits no toxic or hemolytic activity. In vitro, NCTX15 demonstrates XO inhibitory activity. In HUA mice, XO activity is also inhibited, with the 1 mg/kg dose of NCTX15 significantly reducing serum XO levels by 52.6 ± 7.08% [70].

#### 3.1.5. Protein-Rich Processing by-Products

During the production process of frozen cod slices, about half of the body weight is produced as by-products, which can be considered as a protein source for developing bioactive peptides. Five peptides, FF, YF, WPW, WPDARG, and YNVTGW, have been isolated and identified from cod by-product enzymatic hydrolysates. All of these peptides exhibit XO inhibitory activity, and their biological activities have been further confirmed by in vitro synthesis. According to an analysis of the molecular docking data between peptides and XO, these peptides all interact with crucial amino acid residues that impact the activity of XO. In contrast to other peptides, YNVTGW establishes π-π stacking interactions with Phe914 and Phe1009 in addition to its hydrogen bonding connection to Mos3004. It displays the highest XO inhibitory activity due to its wider contact area with XO [71]. The meat industry generates a substantial amount of discarded blood, and within this waste, hemoglobin stands out as a rich source for developing antimicrobial and antioxidant peptides [72]. *Geobacillus stearothermophilus* protease was used to hydrolyze hemoglobin, and following separation, the three XO inhibitory peptides IVYPW, YPWTQ, and LITGLW were identified. IVYPW and YPWTQ forms hydrogen bonds and π-σ interactions with Thr1010 and His875 of XO, respectively, according to molecular docking analysis, which increases the inhibitory activity of XO [73]. Eight food processing protein by-products were hydrolyzed using alkaline serine protease. At a protein concentration of 3 mg/mL, duck hemoglobin hydrolysate exhibits high XO inhibitory activity with an inhibition rate of 84.0% [74]. Feather keratin, one of the best high-quality protein sources, contains many hydrophobic amino acids associated with decreased UA activity [75,76]. As a result, feather keratin may be a new source of anti-hyperuricemic peptides. *Bacillus licheniformis* 8–4 produces the XO inhibitory peptide GNQQVHLQSQDM from feather fermentation broth. It prevents the oxidation of xanthine to UA by occupying the XO active cavity and preventing xanthine from binding to the XO active site. This connection to some of the critical amino acid residues of XO has been revealed by molecular docking [77]. Collagen is the primary structural protein in the skin. Fish skin collagen peptide is widely used in cosmetics, pharmaceuticals, and food. The activity of XO in the liver of mice given fish skin collagen peptide (CP) decreases by 18.19% as compared to that of mice in the HUA model group [78,79]. The main by-product of cold-pressed walnut kernels is degreased walnut meal, which contains >40% protein and is typically used as animal feed or fertilizer. An experiment used a degreased walnut meal to create dephenolized walnut meal hydrolysate (DWMH). The findings revealed that oral administration of DWMH significantly decreases the level of SUA in HUA mice, and the XO inhibitory peptides WPPKN and ADIYTE were later found in the hydrolysate. The inhibition rate of XO by WPPKN (97.95 ± 0.39%) is higher than that of ADIYTE (73.21 ± 0.62%) as a result of the significant interaction between WPPKN and Mo-pt [22]. The pressed cake by-product generated during the oil pressing of sacha inchi is rich in protein, comprising 47–59%. This protein content presents significant potential for the production of protein peptides. In one study, after intragastrically administering sacha inchi oil press-cake protein hydrolysates (SISH), abnormally high XO activity in the serum and liver of HUA mice returned to normal levels. The UA-lowering effect of SISH was partially attributed to its inhibitory activity on XO [80,81]. In conclusion, protein-rich processing by-products also have the bioactivity to lower UA content, which helps to increase the availability of new and potent XO inhibitor manufacturing sources.

#### 3.1.6. Nuts

Peptides from nuts exhibit a variety of biological activities, such as antioxidant, anti-cancer, and antihypertensive effects, which are beneficial for maintaining homeostasis and preventing diseases [82]. In vitro experimental results reveal that walnut protein-derived peptides containing Trp are more potent in inhibiting XO activity compared to peptides lacking Trp. Furthermore, WDQW, which contains two Trp residues, exhibits an inhibitory effect on XO comparable to that of allopurinol. This confirms the connection between the inhibitory activity of peptides on XO and the presence of Trp [75]. *Macadamia integrifolia* antimicrobial protein 2 was virtually hydrolyzed, and the XO inhibitory peptides PGPR, HGGR, and GPY were screened out. All three peptides interact with important XO active cavity amino acid residues, such as Glu802, Leu873, Arg880, Phe914, and Thr1010, and their in vitro XO inhibitory activity is consistent with the findings of molecular docking. In conclusion, these three peptides are promising natural compounds for treating HUA [83].

#### 3.1.7. Rice

As one of the staple foods, rice is also an important source of protein. Some people have recently started using rice peptides as a source of anti-hyperuricemia. In a study, the water extract of shelled *Oryza sativa* fruits was used to create rice-derived peptide-3 (RDP3), whose sequence was determined to be AAAAMAGPK-NH_2_. In vitro, RDP3 has a much lower inhibitory rate on XO (1 mg/mL, 29.45 ± 11.15%) compared to that of allopurinol (10 mg/mL, 99.97 ± 0.44%) [84]. Within a specific range, peptide length reduction can improve cell sensitivity, lower synthesis costs, and preserve the original biological activity of peptides. Rice-derived peptide-1 (RDP1), whose peptide sequence was AAAAGAKAR, was optimized into a short RDP1-M3 (AAAAGA). RDP1-M3 had a greater optimal inhibitory rate to XO than RDP1 did at the same concentration, with values of 39.94 ± 0.44% and 30.57 ± 2.95%, respectively [85,86].

Additionally, 1, 10, and 100 nmol/L RDP1-M3 treatment groups may all lower cell UA levels and XO activity compared to the LO2 cell model group, and the impact is dose-dependent. Cell XO activity dropped to 18.94 ± 0.58, 15.85 ± 0.59, 14.40 ± 0.63 U/gprot, respectively. RDP1-M3 therapy may benefit HUA cells because these findings support its XO inhibition effect [86].

Compared to the 10 mg/kg allopurinol group, the 100 µg/kg and 1 mg/kg RDP1 intragastric injection groups had higher anti-hyperuricemic activity. The relative SUA concentrations of mice were 30.74 ± 1.25 mg/kg, 28.52 ± 1.14 mg/kg, and 24.74 ± 1.21 mg/kg, respectively. RDP1 was found to occupy the Mo-pt of XO and prevent the interaction between XO and xanthine, which decreases the production of UA, according to analysis of molecular docking studies. Another study isolated and discovered a novel rice-derived peptide-2 (RDP2) from water extracts of rice. Its sequence is AAAAGAMPK-NH_2_. Intraperitoneal injection of RDP2 can significantly lower SUA levels by reducing XO activity in the serum and liver of HUA mice [87,88]. The inhibitory activity of RDP3 on XO in vivo was close to that of allopurinol, which is in contrast to the results of in vitro investigations. This may be because RDP3 is degraded into short peptides, which increases its XO inhibitory activity [84].

#### 3.1.8. Legumes

Beyond their edible value, legumes can also serve as raw materials for the preparation of peptides with antioxidant, anti-inflammatory, antihypertensive, and other biological activities [89]. Five peptides with XO inhibitory activity were evaluated by molecular docking kidney bean hydrolysates with XO. At the same concentration (1 mg/mL), synthetic DWYDIK, AVDSLVPIGR, LDNLLR, ISPIPVLK, and ISSLEMTR exhibit XO inhibitory rates of 68.63 ± 5.07%, 55.62 ± 0.29%, 40.20 ± 1.63%, 37.49 ± 1.88%, and 25.08 ± 2.52%, respectively. Among them, DWYDIK has the most remarkable ability to reduce HUA since its inhibitory action on XO is roughly 75% that of febuxostat [90]. The UA content in LO2 cells significantly decreases upon the addition of soy peptides SHECN and SHCMN. Further analysis of the spatial structure of XO indicates that, except for the 10mg/mL SHCMN group, all the other groups exhibit a transformation of the secondary structure from β-turns to α-helices. This results in a more compact XO molecular structure, making it less likely for xanthine to enter the active cavity of XO. This observation provides a solid explanation for the inhibitory activity of these two peptides on XO [91,92].

**Table 1:** Inhibition of XO by peptides derived from different food sources.

Sources	Peptides	Inhibitory Effects	Contact Sites	Interaction Types	Ref.
Pacific bluefin tuna (*Thunnus Orientalis*)	ICRK	IC_50_ = 7.23 mg/mL	Glu802, Glu1261, Phe914, Ala1079, Lys771, Leu648, Thr1010, Val1011, Ser876	Hydrogen bond, salt bridge, carbon-hydrogen bond, unfavorable donor-donor	[51]
	FDAK	IC_50_ = 14.18 mg/mL	Leu648, Asn768, Glu802, Lys771, Phe1009, Phe914, Ala1079, Pro1076	Charge attraction, π-π stacking, carbon-hydrogen bond, hydrogen bond, π-alkyl action	
	MMER	IC_50_ = 16.30 mg/mL	Pro1076, Asn650, Lys771, Glu802, Phe649, Phe1009, Gly647, Leu648	Carbon-hydrogen bond, salt bridge, π-sulfur bond, hydrogen bond	
Skipjack tuna (*Katsuwonus pelamis*)	ACECD	IC_50_ = 7.23 mg/mL	Leu648, Phe649, Asn768, Met770, Lys771, Glu802, Leu873, His875, Ser876, Glu879, Phe914, Phe1009, Thr1010, Val1011, Pro1012, Phe1013, Leu1014, Pro1076, Ala1079, Phe1142	Hydrogen bond, van der Waals force, hydrophobic interaction	[21]
ND	EEAK	IC_50_ = 173.00 ± 0.06 μM	Arg880, Thr1010, Leu648, Lys771, Gln1122, Ser876, Glu802, Phe649, Phe914	Hydrogen bond, carbon-hydrogen bond, salt bridge, charge attraction, π-cation interaction, π-anion interaction	[52]
Round scad (*Decapterus maruadsi*)	KGFP	XO inhibition rate of 5.43 ± 0.20%	Ala1079, Ser1080, Glu802, Phe798	Hydrogen bond, π-π stacking	[53]
	FPSV	XO inhibition rate of 22.61 ± 1.81%	Phe914, Phe1009	π-π stacking	
	FPFP	XO inhibition rate of 20.09 ± 2.41%	Phe914, Phe1009, Phe775	π-π stacking	
	WPDGR	XO inhibition rate of 16.21 ± 0.78%	Phe914, Phe1009	π-π stacking	
*Auxis thazard*	PDL	IC_50_ = 4.37 ± 0.11 mg/mL	Asn768, Ser876, His875, Glu802, Leu873, Lys771, Phe914, Thr803, Pro1076, Thr1010, Arg880, Ala1079, Ala1078, Ala910, Phe1009, Leu1014, Asp872, Phe649, Phe1013, Val1011	Hydrogen bond, van der Waals force, hydrophobic interaction	[55]
	SVGGAL	IC_50_ = 5.59 ± 0.09 mg/mL	Leu648, Asn768, Glu802, Lys771, Phe649, Val1011, Leu1014, Phe914, Phe1009, Leu873, Thr803, Ser876, Phe1013, Met770, Gln1016	Hydrogen bond, van der Waals force, hydrophobic interaction	
Small yellow croaker (*Larimichthys polyactis*)	WDDMEKIW	IC_50_ = 3.16 ± 0.03 mM	Ile1190, Ala1189, Leu744, Gln1201, His579, Val1200, Gly1197, Glu1196, Phe1219, Ile1235, Ile1229, Phe1232, Pro1230, His741, Ala1231, Tyr743, Phe238, Phe742, Tyr592, Met1038, Gly1039, Gly796, Met794, Gly795, Ala582, Gln585, Gln1194, Gly1193	Hydrogen bond, charge attraction, hydrophobic interaction	[56]
	APPERKYSVW	IC_50_ = 5.86 ± 0.02 mM	Arg912, Met1038, Ala582, His579, Gln585, Met794, Gly796, Gly795, Leu744, Tyr743, Tyr592, Gly39, Gln194, Gly193, Gln21, Phe798, Ala1198, Glu1196, Ile1235, Phe1239, Gly1197, Val1200, Phe1232, Ala1231	Hydrogen bond, hydrophobic interaction	
*Ostrea rivularis Gould*	ALSGSW	IC_50_ = 2.17 ± 0.09 mM	Phe1142, Thr1010, Ser876, Tyr1140, Glu879, Leu648, Leu1014, Leu712, Val1011, Leu1014, Pro1076	Hydrogen bond, carbon-hydrogen bond, π-σ interaction, charge attraction	[57]
	GGYGIF	IC_50_ = 4.28 ± 0.43 mM	Asn768, Arg880, Thr1010, Phe914, Leu873, Glu879, Phe649, Ser876, His875, Leu648, Leu712, Val1011, Pro1012, Phe1013, Pro1076, Leu1014, Phe1142	Hydrogen bond, carbon-hydrogen bond, π-hydrogen bond, hydrophobic interaction, π-σ interaction, π-π stacking	
	MAIGLW	IC_50_ = 3.48 ± 0.09 mM	Leu648, Glu879, Tyr1140, His875, Leu873, Phe914, Phe1009, Val1011, Pro1012, Phe1013, Leu1014	Hydrogen bond, carbon-hydrogen bond, hydrophobic interaction	
	AEAQMWR	IC_50_ = 8.85 ± 0.05 mM	Mos3004, Thr1010, Glu80, Glu1261, Arg880	Salt bridge, hydrogen bond, carbon-hydrogen bond, charge attraction	
Pacific white shrimp (*Litopenaeus vannamei*)	AGGINLAR	IC_50_ = 88.51 ± 13.78 mM	Glu802	Hydrogen bond, carbon-hydrogen bond, salt bridge, charge attraction	[59]
	EFGMGGW	IC_50_ = 90.30 ± 2.24 mM	Arg880	Hydrogen bond, carbon-hydrogen bond, charge attraction	
Pacific white shrimp	EDDDA	IC_50_ = 259.46 ± 11.91 mM	Arg880, Val1011, Thr1010, Asn768	Hydrogen bond	[60]
	AGDY	IC_50_ = 21.82 ± 0.15 mM	Asn768, Gul802, Phe914, Lys771	Hydrogen bond, charge attraction	
	GDEY	IC_50_ = 20.67 ± 0.30 mM	Val1011, Thr1010, Ser876, Lys771, Phe914	Hydrogen bond, charge attraction, π-π stacking	
	YGDE	IC_50_ = 47.34 ± 1.55 mM	Asn768, Arg880, Glu802, Phe914	Hydrogen bond, hydrophobic interaction, π-π stacking	
	YNITGW	IC_50_ = 9.78 ± 0.13 mM	Asn768, Glu879, Ser876, His875, Pro1076, Val1011, Leu1014, Leu873, Pro1012, Lys771, Phe914	Hydrogen bond, carbon-hydrogen bond, hydrophobic interaction, charge attraction, π-π stacking	
	PDARG	IC_50_ = 35.01 ± 1.18 mM	Asn768, Glu802, Lys771, Phe914	Hydrogen bond, charge attraction, π-π stacking	
	VTGW	IC_50_ = 118.68 ± 4.84 mM	Thr1010, Asn768, Lys771	Hydrogen bond, hydrophobic interaction	
Bonito	WML	ND	Asn768, Mos3004	Hydrogen bond, π-sulfur bond	[20]
	PGACSN	ND	Glu802, Arg880, Glu1261	Hydrogen bond, charge attraction	
Tuna	FH	IC_50_ = 25.70 mM	Leu648, Phe649, Asn768, Met770, Lys771, Glu802, Thr803, Asp872, Leu873, His875, Ser976, Glu879, Ser876, Arg880, Phe914, Phe1009, Thr1010, Val1011, Phe1013, Leu1014, Ser1075, Pro1076, Ala1078, Ala1079, Try1121	Hydrogen bond, π-π stacking	[54]
Egg	EEK	IC_50_ = 141.00 µmol/L	Lys771, Asn768, Ser876, Glu802, Thr1010, Arg880, Phe1009, His875, Glu879	Carbon-hydrogen bond, salt bridge, hydrogen bond, attractive charge interaction	[62]
Whey	PEW	IC_50_ = 3.46 ± 0.22 mM	Lys771, Asn768, Ser876, Thr1010, Phe1009, Phe914	Hydrophobic interaction, hydrogen bond, π-π stacking	[64]
	LLW	IC_50_ = 3.02 ± 0.17 mM	Phe911, Gln767, Ala1078, Glu802, Gln1040, Phe1009, Phe914	Hydrophobic interaction, hydrogen bond, π-π stacking	
Whey	GL	IC_50_ = 10.20 ± 0.89 mM	Ser876, Arg880, Ser1008, Phe1009, Thr1010, Val1011, Glu802, Ala910, Phe914, Ala1078, Ala1079, Leu648, Leu873, Leu1014, Pro1076, Glu1261	Hydrogen bond, hydrophobic interaction, van der Waals forces	[65]
	PM	IC_50_ = 23.82 ± 0.94 mM	Arg880, Phe914, Glu1261, Glu802, Arg912, Leu648, Gln767, Phe798, Gly799, Leu873, Ser876, Ala910, Phe911, Gly913, Phe1005, Phe1009, Thr1010, Pro1076, Ala1078, Ala1079, Ser1080	Hydrogen bond, hydrophobic interaction, van der Waals forces	
	AL	IC_50_ = 34.49 ± 0.89 mM	Glu802, Arg880, Ala910, Phe914, Ala1078, Gln767, Phe798, Gly799, Leu873, Ser876, Phe911, Arg912, Gly913, Phe1009, Thr1010, Val1011, Leu1014, Pro1076, Thr1077, Ala1079, Ser1080, Glu1261	Hydrogen bond, hydrophobic interaction, van der Waals forces	
	AM	IC_50_ = 40.45 ± 0.92 mM	Arg880, Glu1261, Glu802, Arg912, Gln767, Phe798, Gly799, Leu873, Phe911, Gly913, Phe914, Phe1009, Thr1010, Val1011, Leu1014, Pro1076, Ala1078, Ala1079, Ser1080	Hydrogen bond, hydrophobic interaction, van der Waals forces	
Milk	QLKRFSFRSFIWR	IC_50_ = 6.26 ± 0.03 mM	Phe649, His875	Hydrophobic interaction, hydrogen bond	[66]
	GPVRGPFPIIV	IC_50_ = 4.67 ± 0.24 mM	Glu879	Hydrogen bond	
	VYPFPGPI	IC_50_ = 5.75 ± 0.12 mM	Ser774	Hydrogen bond	
	GFININSLR	IC_50_ = 6.25 ± 0.46 mM	Ser774, Glu879	Hydrogen bond	
	AVFPSIVGR	IC_50_ = 5.04 ± 0.24 mM	Glu879	Hydrogen bond	
	VYPFPGPIPN	IC_50_ = 7.96 ± 0.36 mM	Ser774	Hydrogen bond	
	VYPFPGPIHN	IC_50_ = 8.02 ± 0.35 mM	His875	Hydrogen bond	
	LVYPFPGPIHN	IC_50_ = 6.05 ± 0.03 mM	Ser774, His875	Hydrogen bond	
Pacific cod bone-flesh mixture	FF	IC_50_ = 0.80 mM	Ser876, Thr1010, Phe914, Mos3004	Hydrogen bond, π-π stacking, van der Waals force	[71]
	YF	IC_50_ = 0.52 mM	Asn768, Ser876, Thr1010, Phe914, Phe1009, Mos3004	Hydrogen bond, π-π stacking, van der Waals force	
	WPW	IC_50_ = 1.68 mM	Glu879, Thr1010, Phe914, Phe1009, Mos3004	Hydrogen bond, π-π stacking, van der Waals force	
	WPDARG	IC_50_ = 0.40 mM	Ser710, Glu711, Phe1142	Hydrogen bond	
	YNVTGW	IC_50_ = 0.23 mM	Mos3004, Ser876, Glu879, Thr1010, Glu126	Hydrogen bond, π-π stacking	
Hemoglobin	IVYPW	IC_50_ = 0.63 ± 0.03 mM	Phe1013, Thr1010	π-π stacking, hydrogen bond	[73]
	YPWTQ	IC_50_ = 0.97 ± 0.03 mM	His875	π-cation interaction, π-σ interaction	
	LITGLW	IC_50_ = 1.09 ± 0.03 mM	Phe1142, Glu879	π-σ interaction, Hydrogen bond	
Feather	GNQQVHLQSQDM	IC_50_ = 12.15 mg/mL	Glu802, Asn768, Arg871, His875, Arg880, Phe1009, Thr1010, Glu802, Glu1261	Hydrogen bond, charge attraction	[77]
Dephenolized walnut meal	WPPKN	IC_50_ = 17.75 ± 0.12 mg/mL	Leu1014, Glu802, Phe914, Arg880, Val1011, Phe1013, Asp872, His875, Leu873, Thr1010, Ser876, Glu879	Hydrogen bond	[22]
	ADIYTE	IC_50_ = 19.01 ± 0.23 mg/mL	Leu1014, Glu802, Phe1013, Glu879, Val1011, Asp872, His875, Leu873, Thr803, Ser876, Lys771	Hydrogen bond	
Walnut	PPKNW	ND	Phe649, Leu712, His875, Glu879, Phe883, Pro1012, Phe1013, Tyr1140, Phe1142, Glu1143	Hydrogen bond, hydrophobic interaction, van der Waals force	[75]
	WDQW	ND	Glu802, Leu873, Ser876, Arg880, Phe914, Phe1009, Thr1010, Val1011, Leu1014, Ala1078, Ala1079, Mos3004, Leu648, Phe649, Leu712, His875, Glu879, Pro1012, Phe1013, Pro1076, Phe1142	Hydrogen bond, hydrophobic interaction, van der Waals force	
*Macadamia integrifolia* antimicrobial protein 2	PGPR	IC_50_ = 24.84 ± 0.02 mM	Arg880, Phe914, Thr1010, Leu1014	π-σ interaction, hydrogen bond	[83]
	GPY	IC_50_ = 30.44 ± 0.33 mM	Phe914, Thr1010	π-π stacking, hydrogen bond	
	HGGR	IC_50_ = 24.89 ± 0.19 mM	Glu802, Arg880, Phe914, Thr1010	charge attraction, hydrogen bond	
Rice (*Oryza sativa*)	AAAAMAGPK-NH_2_	ND	Ala1, Ala2, Ala3, Ala4, Ala6, Met5, Pro8, Gly7, Lys9	Hydrogen bond	[84]
	AAAAGAKAR	ND	Asn19, Asp21, Glu232	Hydrogen bond	[86]
Kidney bean	DWYDIK	XO inhibition rate of 68.63 ± 5.07%	Glu802, Lys771, Ser876	Hydrogen bond	[90]
Soy	SHECN	ND	Asp197, Lys178, Gly152, Thr154, Ala150, Arg114, Glu209, Asp206	Hydrogen bond, hydrophobic interaction, π-π stacking	[91]
	SHCMN	ND	Phe1013, Glu802, Phe649, Val1011, Thr1010, Glu879, His875	Hydrogen bond, hydrophobic interaction, π-π stacking	

ND: not determined.

#### 3.1.9. Mushrooms

Edible mushrooms play a crucial role in the treatment of bacterial infections and also serve as a valuable source for producing various bioactive peptides [93]. The strongest XO inhibitory rate was found in Pleurotus ostreatus extract, which was almost eight times stronger than *Lyophyllum cinerascens* extract (10.3 ± 0.4%) when compared to the fruiting body extracts of other five kinds of edible fungi. The identified XO inhibitory peptide Phe-Cys-His can lower SUA levels in HUA mice, and the impact is positively correlated with the dose. According to these findings, Pleurotus ostreatus is a potent bioactive ingredient that might be used to treat HUA [94].

### 3.2. Peptides That Inhibit or Downregulate Key Enzymes in Purine Metabolism

Some food-derived protein peptides can decrease the production of UA in addition to XO activity by downregulating the activities or expression levels of important purine metabolic pathway enzymes. In the presence of inorganic orthophosphate as a second substrate, purine nucleoside phosphorylase (PNP), a widely distributed purine metabolizing enzyme, catalyzes the cleavage of the glycoside bond between ribonucleoside and deoxyribonucleoside to produce purine bases and ribose (deoxyribose)-1-phosphate [95]. By catalyzing the conversion of adenosine to inosine, the essential purine metabolism enzyme ADA controls the levels of adenosine in both intracellular and extracellular space [96]. UA synthesis can be decreased to lessen HUA by inhibiting the activity of these enzymes or reducing their expression levels.

After gastric administration of tuna fish meat oligopeptide (TMOP), the mRNA and protein levels of PNP, ADA, and PRPS in HUA mice decreased, and the effect of reducing UA synthesis in the low-dose group was better than that in the high-dose group [97]. Hydrolysates from *Apostichopus japonicus* (EH-JAP) and *Acaudina leucoprocta* (EH-LEU) reduce the mRNA levels of PRPS and PNP in HUA mice. Moreover, EH-JAP also downregulates the expression levels of ADA in both mRNA and the protein [98,99]. WPH treatment for HUA mice results in a dose-dependent decrease in SUA levels, which is partially attributed to the suppression of ADA activity in the serum and liver. When compared to mice in the HUA model group, mice supplemented with CP exhibit a 26.67% lower level of ADA activity in their livers [68,79]. Various doses of ATO reduce ADA activity in the liver of HUA mice, thereby inhibiting UA synthesis. It is noteworthy that following the administration of a high dose of ATO, the effect closely resembles that of allopurinol [58].

The purine metabolism enzyme xanthine dehydrogenase (XDH) is also involved in the reduction of UA synthesis from xanthine. Instead of O_2_, it uses NAD^+^ as an electron acceptor, unlike in XO [100]. Both RDP2 and SISH actively reduce the expression level of XDH, as well as increase the expression level of uratase, which can promote the degradation of UA into more readily excreted allantoin, and so reduce the concentration of UA [80,88].

### 3.3. Peptides That Regulate the Expression Levels of UA Transporters

Several UA transporters mediate the process of UA excretion. While the expression levels of UA excretion transporters (ABCG2, MRP4, and OAT1) are lower than the normal range in patients with HUA, the expression levels of UA reabsorption transporters (URAT1, OAT4, and GLUT9) are higher than the normal range, leading to abnormal SUA content. According to several researchers, UA excretion can be boosted by restoring the expression levels of these UA transporters (Table 2).

The experiment subsequently investigated whether AEAQMWR, EFGMGGW, and AGGINLAR peptides influence the expression levels in HK-2 cells with HUA because GLUT9 and URAT1 observably affect the reabsorption of UA. The findings showed that three peptide treatments of HK-2 mRNA and protein expression of URAT1 and GLUT9 facilitate UA excretion [59].

A study discovered that following 8 weeks of intragastric administration of EH-JAP and EH-LEU, the mRNA levels of uric acid reabsorption transporters (OAT4, GLUT9, and URAT1) and uric acid secretion transporters (ABCG2, MRP4, and OAT1) in HUA mice were restored [98]. According to the mRNA and protein expression levels, TMOP and CP decreased the levels of URAT1 and GLUT9; however, the former also increased the expression of ABCG2 and the latter regulated OAT1 [79,97]. XO inhibitory peptides CE and KE decrease the production of UA and also have biological effects on GLUT9 and ABCG2 expression levels in terms of mRNA and protein expression [101]. In HUA rats, WPH and peptide PEW downregulate the levels of mRNA and protein expression of URAT1 while upregulating the levels of mRNA and protein expression of OAT1. Additionally, WPH promotes ABCG2 expression at the mRNA level, reversing the tendency in rats toward lowering urine UA [64]. URAT1 and GLUT9 protein expression levels decrease, whereas ABCG2 protein expression levels increase in HUA mice following an anserine gastric injection [102]. At the mRNA level, ATO downregulates the expressions of URAT1 and GLUT9 while upregulating the expressions of ABCG2 and OAT1. This promotes the excretion of UA from the kidneys, thereby reducing the SUA level in HUA mice [58]. In HUA mice, NCTX15 downregulates URAT1 and GLUT9 protein expression levels, and upregulates the OAT1 expression level to promote UA excretion [70].

RDP2 at various injection doses demonstrates biological activity of downregulating the protein expression level of URAT1. An RDP3 (500 μg/kg, 1 mg/kg) intervention also decreases the protein expression level of URAT1 in the kidneys of HUA mice, reducing the SUA content by inhibiting UA reabsorption [87,88]. In HUA mice, RDP1-M3 upregulates the OAT1 protein expression level to promote UA excretion while downregulating URAT1 and GLUT9 protein expression levels [86].

### 3.4. Peptides That Restore Intestinal Flora Composition

Around 20% of UA from food and UA transported from the blood by UA transporters are influenced by the intestinal microbiota. The interaction between the intestinal microbiota and HUA includes regulating the metabolism of purines and the final product UA, producing short-chain fatty acids (SCFAs), or changing the quantity and distribution of UA transporters [103,104]. Experimental data reveal that, in comparison to healthy individuals, the intestinal flora composition of HUA children changes, with a notable drop in the abundance of bacteria that produce sulfur (Bilophila) and SCFAs (Alistipes) [105]. According to a recent study, administering *Lactobacillus plantarum* Q7 to HUA mice can partially repair their disturbed intestinal flora, demonstrating the activity of regulating UA metabolism and reducing HUA [106]. In short, re-establishing the composition of the intestinal flora may be a potent new method for treating HUA. Table 3 summarizes anti-hyperuricemic peptides from food sources that restore the composition of the intestinal flora.

Firmicutes, Bacteroidetes, Proteobacteria, and Actinobacteria are the four phyla that dominate in mice. Both the EH-JAP and EH-LAU groups show a rise in the relative abundance of Bacteroidetes and Firmicutes, and the EH-JAP group restores the relative abundance of Actinobacteria and Proteobacteria. Both groups see increased relative abundance of Clostridia, which can only use purine as an energy source to break down UA, lowering SUA levels. In the EH-LEU group, the abundance of Ruminococcaceae also increases, facilitating UA excretion by generating more SCFAs. The abundance of pathogenic bacteria (Porphyromonas and Bacteroidetes) is decreased. Accordingly, gut microbiota alteration can be viewed as one of the mechanisms by which EH-JAP and EH-LEU relieve HUA [98,107]. Firmicutes and Proteobacteria decrease in the intestinal tracts of HUA mice after an intragastric injection of TMOP, but Bacteroidetes and Actinobacteria increase. The abundance of Bacteroidia, Bacilli, Actinobacteria, and Epsilonproteobacteria is restored at the class level, while the abundance of Clostridia increases much more. The relative abundance of bacteria that produce SCFAs (Clostridium, Ruminococcus, Bifidobacterium, Lachnoclostridium, and Eubacterium) increases due to TMOP, which is remarkable. Along with changes in flora abundance, there are also increases in the concentration of SCFAs (like acetic acid, propionic acid, and butyric acid), which may help reduce HUA by increasing ABCG2 expression and promoting UA excretion [97,108,109]. By gavage treatment of CE and KE, respectively, the relative abundance of Bacteroidetes caused by fructose in the intestines of HUA mice is restored. Furthermore, it has been discovered that the relative abundances of four phyla (Firmicutes, Verrucomicrobia, Proteobacteria, and Actinobacteria) increase in HUA mice. The consumption of dipeptide CE and KE restores the relative abundance of three phyla but further increases the abundance of Verrucomicrobia [101]. Lactobacillus can reduce the absorption of purine in the intestinal tract. Contrary to Lactobacillus, which is widely distributed in the intestinal tract, Saccharomyces cerevisiae secretes urate oxidase that catalyzes UA oxidation to prevent UA accumulation in the body [110,111]. The intestinal flora diversity of HUA mice recovers after 6 weeks of anserine administration, and the abundance of Bacteroidetes and Proteobacteria decreases while the abundance of Firmicutes increases. The abundance of Lactobacillaceae, Clostridiaceae, and Saccharomyces cerevisiae also increases, allowing the intestinal flora to participate generally in purine oxidative metabolism. This suggests that the therapeutic effect of anserine on HUA is partially correlated with the recovery of the intestinal microbiota composition [102]. Different doses of marine fish protein peptide (MFPP) have been found to effectively reduce SUA levels in HUA rats. Upon comparing the species diversity between treatment groups, it was observed that the abundance of beneficial bacteria in HUA rats recovers after MFPP treatment, including Lactobacillus, Blautia, Colidextribacter, and Intestinimonas. The latter three primarily play a role in reducing UA through SCFA production. MFPP demonstrates potential as a therapeutic strategy in dietary interventions to alleviate HUA [112,113,114].

SISH increases butyrate-producing Ruminococcaceae and decreases the number of XO-producing Streptococcus in the intestine, reversing the trend of alterations in the intestinal microbiota in hyperpurine-induced HUA rats. Additionally, after SISH treatment, the probiotics Akkermansia and Alistipes have been shown to increase in the intestinal tract, while Lactobacillus is decreased [80,115,116].

**Table 3:** Food-derived anti-hyperuricemic peptides that restore the composition of the intestinal flora.

Sources	Hydrolysates	Peptides	Flora with Increased Relative Abundance	Flora with Decreased Relative Abundance	Ref.
Sea cucumber	Enzymatic hydrolysates of *Apostichopus japonicus*	NA	*Lactobacillus*, *Lachnospiraceae*, *Clostridiales*	*Porphyromonadaceae*, *Coriobacteriaceae*, *Bacteroides*	[98]
	Enzymatic hydrolysates of *Acaudina leucoprocta*	NA	*Lactobacillus*, *Lachnospiraceae*, *Ruminococcaceae*, *Clostridiales*, *Streptococcus*	*Porphyromonadaceae*, *Bacteroides*	
Tuna	NA	Tuna meat oligopeptides	*Bacteroidetes*, *Actinobacteria*	*Firmicutes*, *Proteobacteria*	[97]
NA	NA	CE	*Bacteroidetes*, *Akkermansia*	*Firmicutes*, *Proteobacteria*, *Actinobacteria*, *Chytridiomycota*, *Escherichia coli*	[101]
	NA	KE	*Bacteroidetes*, *Akkermansia*	*Firmicutes*, *Proteobacteria*, *Actinobacteria*, *Chytridiomycota*	
NA	NA	Anserine	*Firmicutes*, *Lactobacillaceae*, *Clostridiaceae*, *Saccharomyces cerevisiae*, *Roseburia*, *Coprococcus*	*Bacteroidetes*, *Proteobacteria*, *Alcaligenes*, *Lachnoclostridium*	[102]
NA	NA	Marine fish protein peptide	*Lactobacillus*, *Blautia*, *Colidextribacter*, *Intestinimonas*	ND	[112]
Sacha inchi (*Plukenetia volubilis Linneo*)	Sacha inchi oil press-cake protein hydrolysates	NA	*Ruminococcaceae*, *Akkermansia, Alistipes*	*Streptococcus*, *Lactobacillus*	[80]

NA: not applicable. ND: not determined.

Two peptides can be combined to have anti-hyperuricemic effects in addition to one peptide being used alone to treat HUA. HUA mice were given an equal-parts mixture (RCP) of rice peptide (RP) and CP for 14 days in a row. Along with inhibiting XO and ADA function, RCP showed biological activity by upregulating OAT1 at mRNA and protein levels and downregulating URAT1 and GLUT9 at these two expression levels. Simply put, RP and CP worked better together than they did separately to reduce HUA [79].

## 4. Advantages and Challenges of Food-Derived Protein Peptides in the Treatment of HUA

Several studies have discovered bioactive peptides that lower SUA levels in food-derived proteins, including those from animal sources like milk and eggs, those from plant sources like nuts and grains, and even those from protein-rich processing by-products, which can be used as novel sources of anti-hyperuricemic peptides. Peptides have drawn more attention in recent times due to their availability of production when compared to well-known natural XO inhibitors such as phenolic acids and flavonoids [57,117,118]. Importantly, they exhibit relief effects similar to drugs. The inhibitory effects of the identified peptides PGACSN and WML on XO activity increase with concentration, with 20 μM of WML being equivalent to 40 μM of allopurinol [22]. The inhibitory effect of 1 mg/kg RDP1 on XO is higher than that of 10 mg/kg allopurinol in HUA rats. The inhibitory effect of RDP1 on XO is concentration-dependent. In another study, it was found that 1 mg/kg RDP3 exhibits similar effects to a 10 mg/kg allopurinol treatment, reducing XO activity from 32.1 ± 0.4 U/L to 23.4 ± 1.4 U/L [84,87].

When utilizing their biological properties for disease treatment, peptides produced from food proteins pose a low risk of adverse effects [119,120,121]. For instance, the administration of allopurinol in HUA mice results in adverse effects on the kidneys, manifested by renal tubule dilation, the disappearance of the brush boundary on the surface of proximal tubule epithelial cells, and other renal morphological abnormalities [122]. However, treating HUA mice with protein peptides derived from food has not been associated with adverse reactions. However, peptides derived from naturally occurring food proteins are not absolutely safe. Some toxic or allergy-triggering peptide sequences can be encrypted in the food proteins and released after enzymatic hydrolysis [123]. A bioinformatics approach, such as utilizing online databases and machine learning-based prediction servers, is helpful in recognizing these peptides. For example, the AlgPred server can be employed to predict the allergenicity of peptides, while the ToxinPred server can predict their toxicity. To provide a higher level of evidence, some experimental verifications, such as sensitization experiments, the micronucleus test, and Ames test, should be performed. Different proteases have distinct specificities, and it is advisable to choose an appropriate enzyme to prevent the release of these toxic or allergy-triggering peptides. Alternatively, direct synthesis of safe and effective peptides can be pursued.

Although protein peptides from various food sources show great potential in alleviating HUA, as of our current knowledge, there are no commercially available products. The limitation of peptides in the treatment of HUA may be related to their characteristics. For example, peptides are prone to degradation in common oral administration routes, resulting in reduced bioavailability. The charge and polarity of peptides affect their permeability in the enteric membrane, limiting intestinal absorption. Additionally, peptides have a short half-life and require frequent administration to maintain an effective concentration [124,125]. Furthermore, the synthesis of pure peptides primarily employs solid-phase synthesis technology, which is relatively costly. Most importantly, there is a lack of clinical trial data to prove the efficacy of peptides in HUA treatment.

## 5. Conclusions and Future Perspectives

In addition to drug treatment, developing good dietary habits is crucial for the prevention and treatment of HUA. Long-term excessive intake of certain foods, such as seafood and meat with high purine content, beer, and foods rich in fructose, is closely related to abnormal phenomena of high SUA levels, thereby increasing the incidence of HUA. Therefore, the intake of these foods should be strictly controlled. Additionally, consistent consumption of moderate amounts of milk and vitamin-rich fruits and vegetables may help prevent the formation of excessive SUA levels. In recent years, with an increasing emphasis on health, people are more inclined to prevent or treat diseases with natural ingredients. Food protein peptides from a variety of sources have shown biological activity in the treatment of HUA. These food-derived protein peptides exert anti-hyperuricemia bioactivity by inhibiting XO activity, inhibiting or downregulating necessary purine metabolism enzymes, regulating the expression levels of UA transporters, restoring the composition of the intestinal flora, or employing multiple mechanisms.

While various food protein-derived peptides exhibit anti-hyperuricemic activity, most current studies focus on marine fish. In contrast, plant protein peptides have not received enough attention. It is crucial to expand the investigation into additional sources of anti-hyperuricemic peptides, such as protein-rich but low-utilization processing by-products. Additionally, the results of many current studies on HUA alleviation in people are still unknown because they primarily focus on animals or in vitro experiments. More basic research and clinical studies should be conducted to better understand the biological effects and mechanisms of anti-hyperuricemic actions of food-derived protein peptides. Concerning commercialization, the focus should be on improving the production technology and bioavailability of peptides and avoiding the release of toxic or allergy-triggering peptides. If the above-mentioned challenges can be resolved, food-derived protein peptides have many potential applications as functional components for treating HUA.

## Figures and Tables

**Table 2:** Peptides enhance the excretion of UA by restoring the expression levels of UA transporters.

Sources	Hydrolysates	Peptides	Dose	Main Results	Ref.
Pacific white shrimp (*Litopenaeus vannamei*)	NA	AEAQMWR	2 mM	Decrease the mRNA and protein levels of URAT1 and GLUT9.	[59]
	NA	EFGMGGW	2 mM	Decrease the mRNA and protein levels of URAT1 and GLUT9.	
	NA	AGGINLAR	2 mM	Decrease the mRNA and protein levels of URAT1 and GLUT9.	
Sea cucumber	Enzymatic hydrolysates of *Apostichopus japonicus*	NA	150 mg·kg^−1^·d^−1^	Decrease the mRNA levels of OAT4, GLUT9, and URAT1; Increase the mRNA levels of ABCG2, MRP4, and OAT1.	[98]
	Enzymatic hydrolysates of *Acaudina leucoprocta*	NA	150 mg·kg^−1^·d^−1^	Decrease the mRNA levels of OAT4, GLUT9, and URAT1; Increase the mRNA levels of ABCG2, MRP4, and OAT1.	
Tuna	NA	Tuna meat oligopeptides	50, 300 mg·kg^−1^·d^−1^	Decrease the mRNA and protein levels of URAT1 and GLUT9; Increase the mRNA and protein levels of ABCG2.	[97]
Fish skin collagen	NA	Collagen peptide	80 mg·kg^−1^·d^−1^	Decrease the mRNA and protein levels of URAT1 and GLUT9; Increase the mRNA and protein levels of OAT1.	[79]
NA	NA	CE	10 mg·kg^−1^·d^−1^	Decrease the mRNA and protein levels of GLUT9; Increase the mRNA and protein levels of ABCG2.	[101]
	NA	KE	10 mg·kg^−1^·d^−1^	Decrease the mRNA and protein levels of GLUT9; Increase the mRNA and protein levels of ABCG2.	
Whey	Whey protein hydrolyzate	NA	200, 400, 800 mg·kg^−1^·bw	Decrease the mRNA and protein levels of URAT1; Increase the mRNA and protein levels of OAT1; Increase the mRNA level of ABCG2.	[68]
	NA	PEW	30, 60 mg·kg^−1^	Decrease the mRNA and protein levels of URAT1; Increase the mRNA and protein levels of OAT1.	[64]
NA	NA	Anserine	1, 10, 100 mg·kg^−1^·bw	Decrease the protein levels of URAT1 and GLUT9; Increase the protein level of ABCG2.	[102]
*Auxis thazard*	NA	*Auxis thazard* protein	150, 300, 600 mg·kg^−1^·bw	Decrease the mRNA levels of GLUT9 and URAT1; Increase the mRNA levels of ABCG2 and OAT1.	[58]
*Nephila clavata*	NA	QSGHTFK	10,100 μg·kg^−1^, 1 mg·kg^−1^	Decrease the protein levels of URAT1 and GLUT9; Increase the protein level of OAT1.	[70]
Rice (*Oryza sativa*)	NA	AAAAGAMPK-NH_2_	5, 10, 100 μg·kg^−1^	Decrease the protein level of URAT1.	[87]
	NA	AAAAMAGPK-NH_2_	100, 500 μg·kg^−1^, 1 mg·kg^−1^	Decrease the protein level of URAT1.	[84]
	NA	AAAAGA	1 mg·kg^−1^	Decrease the protein levels of URAT1 and GLUT9; Increase the protein level of OAT1.	[86]

NA: not applicable.

## Data Availability

Data are contained within the article.

## Abbreviation List

Uric acid: UA; serum uric acid: SUA; hyperuricemia: HUA; xanthine oxidase: XO; urate transporter 1: URAT1; phosphoribosyl pyrophosphate synthetase: PRPS; hypoxanthine phosphoribosyltransferase: HPRT; inosine monophosphate: IMP; guanosine monophosphate: GMP; organic anion transporter 4: OAT4; glucose transporter 9: GLUT9; urate transporter: UAT; multidrug resistance-associated protein 4: MRP4; ATP-binding transporter G family member 2: ABCG2; bonito hydrolysates: BH; tuna protein hydrolysates: TPH; *Auxis thazard* oligopeptides, ATO; whey protein hydrolysates: WPH; collagen peptide: CP; dephenolized walnut meal hydrolyzates: DWMH; sacha inchi oil press-cake protein hydrolyzates: SISH; rice-derived peptide-3: RDP3; rice-derived peptide-1: RDP1; rice-derived peptide-2: RDP2; purine nucleoside phosphorylase: PNP; adenosine deaminase: ADA; tuna fish meat oligopeptide: TMOP; hydrolysates of *Apostichopus japonicus*: EH-JAP; hydrolysates of *Acaudina leucoprocta*: EH-LEU; xanthine dehydrogenase: XDH; short-chain fatty acids: SCFAs; marine fish protein peptide, MFPP; the equal-parts mixture of rice peptide and collagen peptide: RCP; ATP: adenosine triphosphate; ADP: adenosine diphosphate; AMP: adenosine monophosphate; XMP: xanthosine monophosphate.
